# Wisteria floribunda agglutinin-positive Mac-2-binding protein as a diagnostic biomarker in liver cirrhosis: an updated meta-analysis

**DOI:** 10.1038/s41598-020-67471-y

**Published:** 2020-06-29

**Authors:** Shu Feng, Zhonghao Wang, Yanhua Zhao, Chuanmin Tao

**Affiliations:** Department of Laboratory Medicine, West China Hospital, Sichuan University, Chengdu, 610041 Sichuan China

**Keywords:** Diagnostic markers, Liver fibrosis, Tumour biomarkers

## Abstract

Wisteria floribunda agglutinin-positive Mac-2-binding protein (WFA+-M2BP) had been suggested as a possible glycobiomarker for assessing liver fibrosis. Here, we conducted this updated meta-analysis to systematically investigate the predictive accuracy of WFA+-M2BP for diagnosing liver fibrosis and hepatocellular carcinoma (HCC) by comparing with multiple non-invasive indicators. We searched relevant literatures from Pubmed, Web of Science, EMBASE and Cochrane Library and enrolled 36 eligible studies involving 7,362 patients. Summary results were calculated using bivariate random effects model. The pooled sensitivities, specificities and areas under the summary receiver operating characteristic curves (AUSROCs) of WFA+-M2BP for identifying mild fibrosis, significant fibrosis, advanced fibrosis, cirrhosis, and HCC were 0.70/0.68/0.75, 0.71/0.75/0.79, 0.75/0.76/0.82, 0.77/0.86/0.88, and 0.77/0.80/0.85, respectively. The accuracy of WFA+-M2BP was strongly affected by etiology and it was not better than other non-invasive indicators for predicting early fibrosis. It showed similar diagnostic performance to hyaluronic acid and FibroScan for cirrhosis, but was equivalent to α-fetoprotein for HCC. In conclusion, WFA+-M2BP was suitable to diagnose late stage of liver fibrosis, especially cirrhosis. Individual cutoff value of WFA+-M2BP could be used to grade liver fibrosis in different etiology. Combined diagnostic model was suggested to improve its predictive accuracy for HCC.

## Introduction

Chronic liver diseases (CLDs) are major health problems that cause significant economic burdens worldwide^1^. Globally, CLDs affect 360 per 100,000 persons, cause more than 1.75 million deaths annually, and are ranked as the 12th leading cause of deaths^1,2^. A wide range of etiologies, which include viral hepatitis (hepatitis B and C), alcoholic liver disease (ALD), nonalcoholic fatty liver disease (NAFLD), nonalcoholic steatohepatitis (NASH), autoimmune hepatitis (AIH), primary biliary cirrhosis (PBC), biliary atresia (BA), and other metabolic disorders, can cause CLDs^3,4^. In CLDs, liver fibrogenesis could be initiated due to dysfunctions of multiple cell types: (1) in diseases with bile ductular proliferation (i.e. PBC, BA), destruction of cholangiocytes leads to loss of bile ducts, resulting in cholestasis followed by hepatic damage and hepatic failure^5,6^; (2) in viral hepatitis (i.e. hepatitis B, hepatitis C), quiescent hepatic stellate cells (HSCs) can be activated by cytokines and chemokines secreted from infected hepatocytes or virus-exposed Kupffer cells, causing the deposition of extracellular matrix (ECM)^7^; (3) in NAFLD and NASH, excessive lipid accumulation promotes lipotoxicity, triggering hepatocytes death and inflammation^8^. Continuous destruction and regeneration of hepatocytes could generate distortions of normal hepatic architecture and replication-related mutations, bringing about liver cirrhosis and, even worse, hepatocellular carcinoma (HCC)^9,10^.

The severity of liver fibrosis is an important aspect for the management of patients with CLDs, both for predicting clinical outcomes and guiding therapies^11,12^. Liver biopsy remains the gold standard for the accurate assessment of fibrosis. However, its use is limited due to its invasiveness. Imaging technologies such as ultrasonography, magnetic resonance imaging, hepatic arteriography, and transient elastography had also been developed and widely used for diagnosis of liver fibrosis and HCC. For example, FibroScan is an imaging-based method that has been most studied^13^. In recent years, with the growing interest in the use of non-invasive methods for accurate assessment of fibrosis, serum biomarkers such as hyaluronic acid (HA), procollagen III, platelet count (PLT), Fibrotest, aspartate aminotransferase-to-platelet ratio index (APRI), and fibrosis-4 index (FIB-4) had been discussed^2,14^.

More recently, the possible use of Wisteria floribunda agglutinin-positive Mac-2-binding protein (WFA+-M2BP) as a novel non-invasive serum biomarker to predict disease severity of CLDs had also been suggested. Mac-2-binding protein (M2BP) is a secretory glycoprotein which contains seven *N*-glycans per monomer^15^. In the serum, 10–16 monomers of M2BP form a “doughnut-shaped” polymer which presents 70–112 *N*-glycans^16^. It has been found that alterations of M2BP happen during the progression of liver disease and fibrosis due to the changes in N-glycosylation (i.e. sialylation or extension of polylactosamine)^16^, however, the underlying mechanism is unclear. As a robust lectin that binds the GalNAc residue of *N*-glycans and *O*-glycans and the clustered LacNAc structure, Wisteria floribunda agglutinin (WFA) can recognize the altered *N*-glycans of M2BP specifically^17^. Thus, this specific glycoprotein was described as WFA+-M2BP and renamed as M2BPGi (Mac-2-binding protein glycosylation isomer) after the commercialization of the diagnostic reagent. In liver, WFA+-M2BP could promote fibrogenesis by acting as an important messenger between HSCs (secrete WFA+-M2BP) and Kupffer cells (express the ligand of M2BP, Mac-2)^18,19^. Additionally, WFA+-M2BP could be increased by TGF-β1 in LX-2 cell and it correlates with serum IP-10 and sICAM-1 levels in patients with AIH^20,21^. Those evidences suggested serum WFA+-M2BP has a great potential to serve as a biomarker for reflecting the liver status of CLD patients ^16,22^.

The clinical application of WFA+-M2BP has been widely promoted after Japanese public health insurance supported its diagnosis expense since 2015^15^. Increased publications have been carried out to estimate the clinical performance of WFA+-M2BP. One meta-analysis reported the possible utility of WFA+-M2BP for liver fibrosis staging caused by various etiologies^23^, however, it did not compare the predictive accuracy of WFA+-M2BP with multiple widely used non-invasive biomarkers. To systematically examine the performance of WFA+-M2BP for diagnosing liver fibrosis and HCC, we conducted an updated meta-analysis by including more literatures and more evaluation parameters. Our results indicated that WFA+-M2BP could be a satisfactory biomarker for staging cirrhosis, and its combined use with AFP may further improve its predictive accuracy for HCC. By elaborating on the advantages and limitations of its diagnostic accuracy in liver fibrosis and HCC, we hope our study could help clinicians make cautious and accurate diagnosis.

## Results

### Basic characteristics of studies

**Figure 1:**
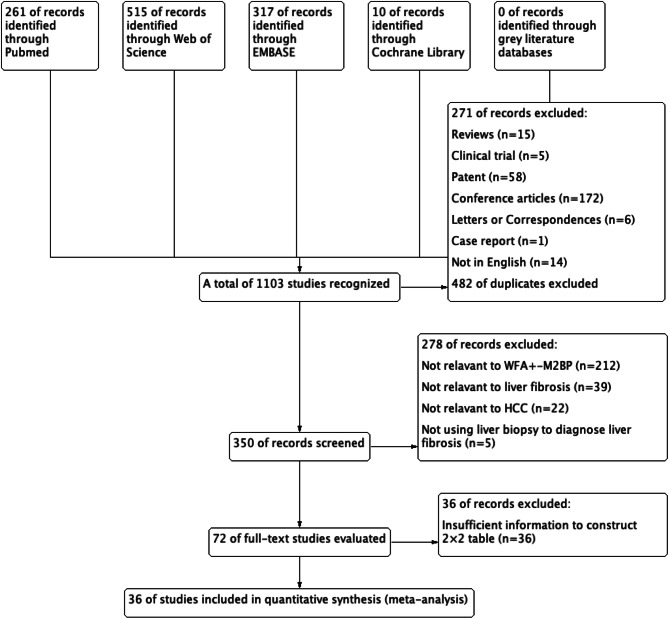
The flow chart of the meta-analysis conducted.

As shown in Fig. 1, after excluding duplicates and non-experimental studies, 350 references were identified. Full-text review on 72 original articles eligible for detailed evaluation were conducted after we excluded non-relevant references. A total of 36 articles were further removed because of insufficient information to construct a 2 × 2 table. Ultimately, the remaining 36 articles were selected for meta-analysis.

We listed the main features of the included studies in Table 1 and 2. Overall, 7,362 participants were included. Among the 36 included articles, 29 articles studied the diagnostic accuracy of WFA+-M2BP on liver fibrosis and 8 articles were on HCC. For the studies on fibrosis, we noticed that 3 articles enrolled both training group and validation group^24–26^, and 2 articles recruited patients with 2 different etiologies^27,28^. Thus, we considered them as individual studies when the calculation of diagnostic accuracy was conducted. Overall, 7 kinds of etiologies of liver fibrosis that include HBV (n = 12), HCV (n = 10), NAFLD (n = 3), NASH (n = 3), AIH (n = 1), BA (n = 2), and PBC (n = 2), as well as mixed etiologies (n = 1) were discussed. HCC was mainly caused by 3 etiologies here: HBV, HCV and NAFLD. All studies employed retrospective design and used lectin-Ab sandwich immunoassay to detect serum WFA+-M2BP levels.

**Table 1:** Characteristics of the included studies on liver fibrosis.

Study	Region	Number of patients	Diagnostic indicators	Median age (year)	Male %	Etiology	Histological system	Blind	Liver biopsy length (mm)	Interval between biopsy and blood test	Fibrosis (0-1/2/3/4)	WFA+-M2BP optimal cutoff values (COI)
Kuno 2013^16^	Japan	160	WFA+-M2BP & FIB-4 & HA	54.9	26.9	HCV	Unclear	Yes	Unclear	Unclear	66/41/33/20	NA
Abe 2015^29^	Japan	289	WFA+-M2BP & APRI & AST/ALT & FIB-4 & HA & PLT	54.8	55.0	NAFLD	Brunt	Yes	> 15	Same day or within 2 months	148/49/41/51	≥ F1: 0.59; ≥ F2: 0.90; ≥ F3: 0.94; F4: 1.46
Toshima 2015^22^	Japan	200	WFA+-M2BP & APRI & HA	64.0	67.5	Mixed (HBV, HCV, alcoholism, and non-infection)	METAVIR	Yes	Unclear	Same day	129/21/16/34	≥ F1: 1.00; ≥ F2: 1.86; ≥ F3: 2.21; F4: 2.64
Umemura 2015^30^	Japan	137	WFA+-M2BP & APRI & AST/ALT & FIB-4	57.0	19.0	PBC	METAVIR	Yes	> 15	Same day	81/27/18/11	≥ F1: 0.70; ≥ F2: 1.00; ≥ F3: 1.40; F4: 2.00
Heo 2016^31^	South Korea	95	WFA+-M2BP	51.0	72.6	HBV	Batts and Ludwig	Yes	Unclear	Same day	16/29/10/40	≥ F2: 0.80; ≥ F3: 1.60; F4: 2.00
Ichikawa 2016^32^	Japan	112	WFA+-M2BP & APRI & FIB-4 & HA & PLT	47.0	64.3	HBV	Revised Inuyama	Yes	> 15	Same day	40/26/24/22	≥ F2: 0.94; ≥ F3: 1.26; F4: 1.26
Ishii 2016^33^	Japan	189	WFA+-M2BP & APRI & AST/ALT& FIB-4 & HA & PLT	44.0	62.4	HBV	METAVIR	Unclear	Unclear	Unclear	108/37/28/16	≥ F2: 1.40; ≥ F3: 1.40; F4: 1.90
Nishikawa_a 2016^2^	Japan	84	WFA+-M2BP & APRI & AST/ALT & FIB-4 & HA & PLT	64.0	17.9	AIH	METAVIR	No	Unclear	Unclear	18/24/24/18	≥ F3: 3.70; F4: 3.90
Nishikawa_b 2016^34^	Japan	57	WFA+-M2BP & APRI & FIB-4 & HA & PLT	59.0	14.0	PBC	METAVIR	Unclear	Unclear	Unclear	24/17/11/5	≥ F3: 3.40; F4: 3.70
Nishikawa_c HBV 2016^27^	Japan	249	WFA+-M2BP & APRI & FIB-4 & HA & PLT	45.6	62.2	HBV	METAVIR	Unclear	Unclear	Unclear	138/51/41/19	≥ F2: 1.37; ≥ F3: 1.42; F4: 1.86
Nishikawa_c HCV 2016^27^	Japan	386	WFA+-M2BP & APRI & FIB-4 & HA & PLT	60.9	46.6	HCV	METAVIR	Unclear	Unclear	Unclear	111/63/90/122	≥ F2: 2.42; ≥ F3: 2.03; F4: 2.42
Nishikawa_d 2016^35^	Japan	134	WFA+-M2BP & APRI & FIB-4 & HA & PLT	51.7	48.5	NASH	Brunt	Unclear	Unclear	Unclear	28/68/25/13	≥ F2: 1.00; ≥ F3: 1.10; F4: 1.60
Nishikawa_e Tr 2016^24^	Japan	125	WFA+-M2BP & APRI & FIB-4 & HA & PLT	45.9	59.2	HBV	METAVIR	Unclear	Unclear	Unclear	73/27/14/11	≥ F3: 1.42
Nishikawa_e Va 2016^24^	Japan	124	WFA+-M2BP & APRI & FIB-4 & HA & PLT	45.3	65.3	HBV	METAVIR	Unclear	Unclear	Unclear	65/24/27/8	≥ F3: 1.42
Nishikawa_f Tr 2016^25^	Japan	210	WFA+-M2BP & APRI & AST/ALT & FIB-4 & HA & PLT	59.9	49.0	HCV	Unclear	Unclear	> 15	Unclear	70/34/46/60	≥ F3: 1.82
Nishikawa_f Va 2016^25^	Japan	176	WFA+-M2BP & APRI & FIB-4 & HA & PLT	62.2	43.8	HCV	Unclear	Unclear	> 15	Unclear	41/29/44/62	≥ F3: 1.82
Shigefuku_NAFLD 2016^28^	Japan	58	WFA+-M2BP & APRI & AST/ALT & FIB-4 & HA & PLT	NA	NA	NAFLD	Brunt	Yes	> 15	Same day	NA	≥ F3: 1.06
Shigefuku_HCV 2016^28^	Japan	72	WFA+-M2BP & APRI & AST/ALT & FIB-4 & HA & PLT	NA	NA	HCV	Desmet	Yes	> 15	Same day	NA	≥ F3: 3.28
Ura 2016^36^	Japan	146	WFA+-M2BP	NA	44.5	HCV	METAVIR	Unclear	Unclear	Unclear	91/18/19/18	≥ F2: 2.14; F3: 2.17
Yamada 2016^37^	Japan	64	WFA+-M2BP & APRI & HA	1.1	25.0	BA	METAVIR	Unclear	Unclear	Same day	1/1/11/51	F4: 3.53
Zou Tr 2016^26^	China	221	WFA+-M2BP & APRI & AST/ALT & FIB-4	38.0	68.3	HBV	METAVIR	Unclear	Unclear	Same day	132/42/23/24	≥ F2: 1.06
Zou Va 2016^26^	China	76	WFA+-M2BP	37.0	61.8	HBV	METAVIR	Unclear	Unclear	Same day	39/17/10/10	≥ F2: 1.06
Huang 2017^38^	Taiwan	229	WFA+-M2BP	52.8	52.8	HCV	METAVIR	Yes	> 15	Unclear	85/56/38/50	≥ F1:1.42; ≥ F2:1.61; ≥ F3:1.42; F4:2.67
Lai 2017^39^	Malaysia	220	WFA+-M2BP	50.1	51.8	NASH	Unclear	Yes	Unclear	Same day	161/16/36/7	≥ F1:0.57; ≥ F2:0.66; ≥ F3:0.69; F4:0.7
Noguchi 2017^40^	Japan	70	WFA+-M2BP & APRI & FIB-4 & PLT	48.6	52.87	HBV	METAVIR	Unclear	Unclear	Same day	34/17/13/6	≥ F2:0.81; ≥ F3: 0.82
Fujita 2018^41^	Japan	122	WFA+-M2BP & APRI & FIB-4	53	67.2	HCV	METAVIR	Unclear	Unclear	Unclear	27/66/20/9	≥ F3: 2.19
Jekarl 2018^42^	South Korea	151	WFA+-M2BP & APRI & FIB-4 & FibroScan	44.6	66.9	HBV	Knodell	Unclear	> 15	Same day	8/86/42/15	≥ F3:0.76; F4: 0.71
Mak 2018^43^	China	327	WFA+-M2BP	38.1	70.0	HBV	Ishak	Yes	> 10	Within 90 days	292/206/50/6	≥ F2:0.25; ≥ F3:0.45; F4:0.96
Matsuura 2018^44^	Japan	84	WFA+-M2BP & APRI & FIB-4 & FibroScan	63	57.1	HCV	METAVIR	Unclear	Unclear	Unclear	20/20/19/25	≥ F4: 2.66
Ogawa 2018^45^	Japan	165	WFA+-M2BP & APRI & AST/ALT & FIB-4 & FibroScan	54.2	58.2	NAFLD	Brunt	Unclear	> 15	Same day or within 2 months	83/24/47/11	≥ F2: 0.83
Ueno 2018^46^	Japan	37	WFA+-M2BP & APRI & AST/ALT & FIB-4 & PLT	18	32.4	BA	METAVIR	Unclear	Unclear	Unclear	16/6/4/11	≥ F2:1.59; ≥ F3:1.67; F4:1.84
Kanno 2019^47^	Japan	85	WFA+-M2BP & APRI & FIB-4 & HA & PLT	NA	47.1	NASH	Brunt	Unclear	Unclear	Within 1 month	12/14/31/28	≥ F4: 3.11
Yeh 2019^48^	Taiwan	160	WFA+-M2BP	40	76.9	HBV	METAVIR	Unclear	Unclear	Same day	72/37/25/26	≥ F1:0.96; ≥ F2:1.345; ≥ F3:1.535; F4:1.665
Nagata 2016^49^	Japan	108	WFA+-M2BP & APRI & FIB-4 & HA	NA	NA	HCV	Desmet	Unclear	Unclear	Unclear	13/49/25/21 (F0/1/2/3–4)	≥ F3: 2.2

WFA+-M2BP, wisteria floribunda agglutinin-positive Mac-2-binding protein; APRI, Aspartate aminotransferase-to-platelet ratio index; FIB-4, Fibrosis-4 index; AST/ALT, AST to ALT ratio; HA, hyaluronic acid; PLT, platelet count; HBV, hepatitis B virus; HCV, hepatitis C virus; NAFLD, nonalcoholic fatty liver disease; NASH, nonalcoholic steatohepatitis; AIH, autoimmune hepatitis; PBC, primary biliary cirrhosis; BA, biliary atresia; COI, cutoff index; CI, confidence interval; PLR, positive likelihood ratio; NLR, negative likelihood ratio; DOR, diagnostic odds ratio; AUSROC, area under the summary receiver operating characteristic curve; Tr, training group; Va, validation group; NA, not available.

**Table 2:** Characteristics of the included studies on HCC.

Study	Region	Number of all participates	Number of HCC patients	Diagnostic indicators	Median age (year)	Male %	Etiology	WFA+-M2BP optimal cutoff values (COI)
Nagata 2016^49^	Japan	119	8	WFA+-M2BP & APRI & FIB-4 & AFP	NA	58.8	HCV	2.4
Cheung 2017^50^	China	114	57	WFA+-M2BP	NA	NA	HBV	0.69
Chuaypen 2018^51^	Japan	30	150	WFA+-M2BP & AFP	NA	75.7	HBV	2.4
Kawanaka 2018^52^	Japan	331	51	WFA+-M2BP	NA	51.4	NAFLD	1.255
Lin 2018^53^	Taiwan	921	122	WFA+-M2BP & AFP	NA	NA	HCV	1.5
Kim 2019^54^	South Korea	170	64	WFA+-M2BP	55	77.6	HBV	2.14
Mak_a 2019^55^	China	207	14	WFA+-M2BP	40	57.0	HBV	0.685
Mak_b 2019^56^	China	78	39	WFA+-M2BP	NA	NA	HBV	1.15

HCC, hepatocellular carcinoma; WFA+-M2BP, wisteria floribunda agglutinin-positive Mac-2-binding protein; APRI, Aspartate aminotransferase-to-platelet ratio index; FIB-4, Fibrosis-4 index; AFP, α-fetoprotein; HBV, hepatitis B virus; HCV, hepatitis C virus; NAFLD, nonalcoholic fatty liver disease; COI, cutoff index, NA, not available.

### Quality assessment

On the basis of QUADAS-2 assessment, the overall quality of included studies was moderate. As shown in Supplementary Figs. 1, 2, in terms of patient selection, 13 studies had high risk of bias because of inappropriate exclusions or case–control designs. A total of 29 studies had high risk of bias in index test because of the awareness of reference standard result before conducting the index test. Five studies did not mention the use of blind method for index tests when explaining the reference standard results. Regarding flow and timing, 25 studies had high or unclear risk of bias because not all patients received the same reference standard or due to unclear interval between index test and reference standard. Moreover, we had significant concerns on 7 studies when evaluate the applicability of their patient selections.

**Figure 2:**
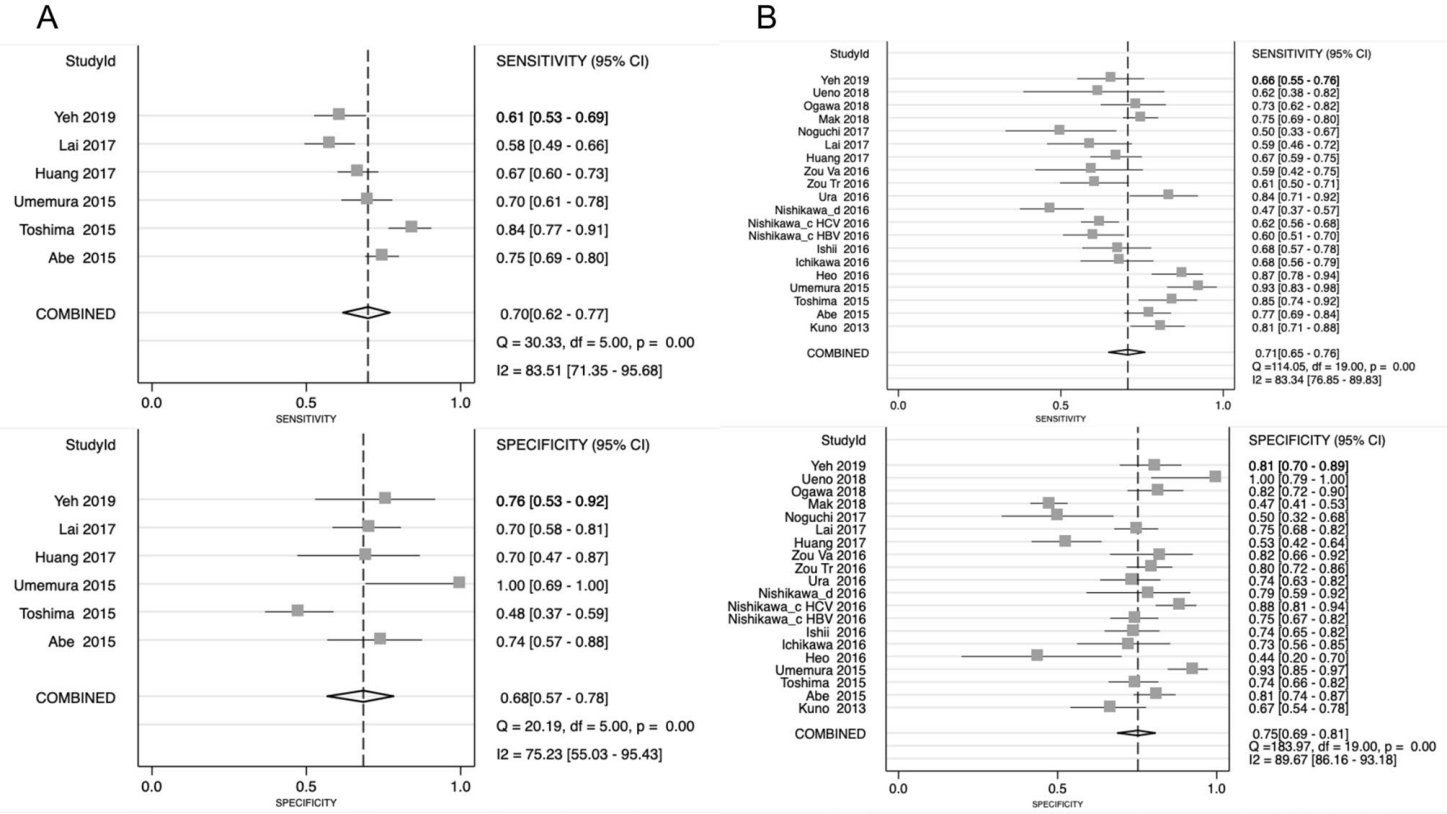
Forest plots of sensitivity and specificity of WFA+-M2BP for the diagnosis of mild fibrosis (**A**) and significant fibrosis (**B**).

### Pooled predictive accuracy of WFA+-M2BP in liver fibrosis

**Figure 3:**
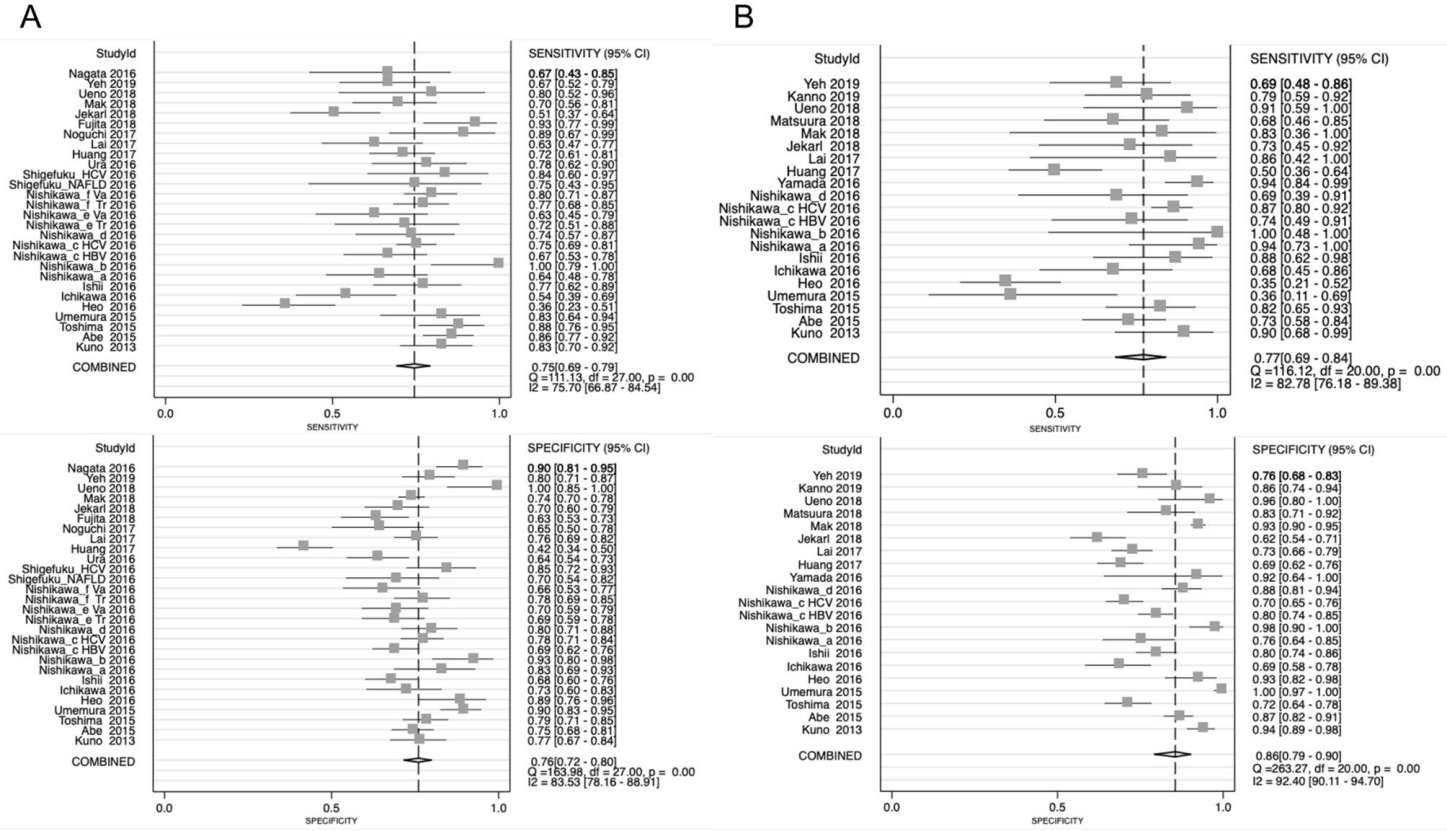
Forest plots of sensitivity and specificity of WFA+-M2BP for the prediction of advanced fibrosis (**A**) and cirrhosis (**B**).

Here, we summarized the predictive accuracy of WFA+-M2BP in each liver fibrosis. A total of 6 studies with 1,235 patients were evaluated for the performance of WFA+-M2BP on predicting mild fibrosis. The pooled sensitivity and specificity were 0.70 (95% CI 0.62–0.77) and 0.68 (95% CI 0.57–0.78), respectively (Fig. 2A). Besides, the pooled AUSROC was 0.75 (95% CI 0.71–0.78). Twenty studies with 3,602 patients were included in significant fibrosis. The pooled sensitivity, specificity and AUSROC were 0.71 (95% CI 0.65–0.76), 0.75 (95% CI 0.69–0.81), and 0.79 (95% CI 0.75–0.82), respectively (Fig. 2B). For predicting advanced fibrosis, 28 studies involving 4,427 patients were assessed. The pooled sensitivity, specificity and AUSROC were 0.75 (95% CI 0.69–0.79), 0.76 (95% CI 0.72–0.80), and 0.82 (95% CI 0.78–0.85), respectively (Fig. 3A). For cirrhosis, 21 studies with 3,449 patients were identified. As displayed in Fig. 3B, the pooled sensitivity and specificity were 0.77 (95% CI 0.69–0.84) and 0.86 (95% CI 0.79–0.90), respectively. The pooled AUSROC was 0.88 (95% CI 0.85–0.91). Those pooled results demonstrated that the predictive accuracy of WFA+-M2BP greatly increased with the progression of liver fibrosis. Its level could nicely reflect the presence of late fibrosis especially cirrhosis. High AUSROC indicated WFA+-M2BP could be applied as an alternative biomarker for biopsy when diagnosing cirrhosis.

### Heterogeneity analysis, threshold effect and meta-regression

To investigate the heterogeneities of the included studies, threshold effect and overall heterogeneity were analyzed. Significant heterogeneities existed in each stage of liver fibrosis (Q = 23.11, I^2^ = 91%, P < 0.001; Q = 94.75, I^2^ = 98%, P < 0.001; Q = 50.32, I^2^ = 96%, P < 0.001; Q = 64.79, I^2^ = 97%, P < 0.001). However, no significant threshold effect was found. In four liver fibrosis stages from mild fibrosis to cirrhosis, the spearman correlation coefficients between sensitivities and specificities were − 0.94 (P = 0.88), − 0.01 (P < 0.01), − 0.02 (P < 0.01), and − 0.16 (P = 0.03), respectively.

Meta-regression analysis (at least 10 studies were requested) was performed to further discuss the cause of heterogeneity in the studies reported for significant fibrosis, advanced fibrosis, and cirrhosis. We investigated 10 factors that might be the potential sources of heterogeneity: year of publication, region, median age, male proportion, number of patients, etiology, histological system, liver biopsy length, interval between biopsy and blood test, and blind method. For identifying significant fibrosis, the accuracy of WFA+-M2BP could be influenced by age, male proportion, etiology, and blind method (P < 0.01, P = 0.07, P = 0.05, and P < 0.01, respectively). For advanced fibrosis, the performance of WFA+-M2BP could be affected by age, male proportion, etiology, and region (P < 0.01, P < 0.01, P = 0.01, and P < 0.01, respectively). For cirrhosis, the heterogeneity of WFA+-M2BP for the detection might be due to the heterogeneity of age, male proportion, region, etiology, and blind method (P < 0.01, P < 0.01, P = 0.02, P = 0.07, and P = 0.08, respectively).

### Predictive accuracy of WFA+-M2BP in liver fibrosis stratified by etiology

As etiology was one of the main reasons of heterogeneities based on our meta-regression analysis, we further analyzed the predictive accuracy of WFA+-M2BP in liver fibrosis caused by various etiologies. We combined studies related to NAFLD and NASH together, and combined studies related to AIH, BA, PBC and mixed etiologies into the “other etiologies” category because of limited number of references. Intriguingly, WFA+-M2BP showed diverse diagnostic accuracies in different etiology groups (Table 3). In general, for the prediction of significant fibrosis, advanced fibrosis, and cirrhosis, WFA+-M2BP owned the best diagnostic accuracies among patients with AIH, BA, PBC or mixed etiologies by reaching the highest pooled sensitivity, specificity, PLR, DOR, AUSROC and lowest NLR, when the results were compared with patients in other etiology groups. Besides, for advanced fibrosis, heterogeneities dramatically dropped in different etiology groups except for HBV and HCV. And for cirrhosis, no heterogeneity was found in the subgroup of NAFLD or NASH (Q = 3.11, I^2^ = 36%, P = 0.106), indicating the accuracy of WFA+-M2BP was influenced by the etiology of disease. In Table 3, different weighted mean WFA+-M2BP values were observed in different etiologies, suggesting individual cutoff value of WFA+-M2BP should be applied to grade liver fibrosis in each etiology. In addition, we noticed that compared with significant fibrosis and advanced fibrosis, WFA+-M2BP possessed the highest AUSROCs in diagnosing cirrhosis regardless of the etiology.

**Table 3:** Overview of meta-analyses results for liver fibrosis stratified by etiology.

Fibrosis stages	Number of studies	Number of patients	Weighted Mean WFA+-M2BP value (COI)	Overall Heterogeneity		Pooled sensitivity (95% CI)	Pooled specificity (95% CI)	Pooled PLR (95% CI)	Pooled NLR (95% CI)	Pooled DOR (95% CI)	Pooled AUSROC (95% CI)
				Q value, P value	I^2^ (%)						
**HBV**											
Significant fibrosis	9	1,499	0.97	60.36, < 0.001	97	0.67 (0.61–0.73)	0.68 (0.58–0.77)	2.1 (1.6–2.7)	0.48 (0.43–0.53)	4 (3–6)	0.72 (0.68–0.76)
Advanced fibrosis	10	1,602	1.14	14.41, < 0.001	86	0.65 (0.55–0.73)	0.73 (0.69–0.76)	2.4 (2.1–2.7)	0.49 (0.39–0.61)	5 (4–7)	0.75 (0.71–0.79)
Cirrhosis	7	1,283	1.43	19.75, < 0.001	90	0.67 (0.52–0.79)	0.82 (0.72–0.89)	3.7 (2.5–5.4)	0.40 (0.28–0.58)	9 (5–16)	0.81 (0.77–0.84)
**HCV**											
Significant fibrosis	4	921	2.12	25.71, < 0.001	92	0.73 (0.63–0.81)	0.72 (0.57–0.84)	2.6 (1.6–4.2)	0.37 (0.27–0.52)	7 (4–14)	0.79 (0.75–0.82)
Advanced fibrosis	9	1,609	1.98	20.67, < 0.001	90	0.78 (0.74–0.81)	0.73 (0.63–0.81)	2.8 (2.0–4.0)	0.31 (0.25–0.38)	9 (6–15)	0.78 (0.74–0.82)
Cirrhosis	4	859	2.53	13.20, 0.001	85	0.78 (0.57–0.90)	0.81 (0.67–0.91)	4.2 (1.9–9.0)	0.28 (0.12–0.63)	15 (3–68)	0.87 (0.83–0.89)
**NAFLD or NASH**											
Significant fibrosis	4	808	0.84	4.12, 0.064	51	0.65 (0.52–0.76)	0.78 (0.73–0.83)	3.0 (2.1–4.3)	0.45 (0.30–0.65)	7 (3–14)	0.80 (0.76–0.83)
Advanced fibrosis	4	701	0.90	2.88, 0.119	30	0.76 (0.65–0.85)	0.76 (0.72–0.79)	3.1 (2.6–3.8)	0.32 (0.21–0.48)	10 (6–17)	0.77 (0.73–0.81)
Cirrhosis	4	728	1.45	3.11, 0.106	36	0.76 (0.64–0.85)	0.84 (0.77–0.89)	4.7 (3.3–6.8)	0.28 (0.19–0.43)	17 (9–30)	0.85 (0.82–0.88)
**Other etiologies**^a^											
Significant fibrosis	3	374	1.52	10.47, 0.003	81	0.83 (0.72–0.90)	0.92 (0.75–0.98)	10.8 (3.1–37.3)	0.18 (0.11–0.31)	58 (16–207)	0.93 (0.90–0.95)
Advanced fibrosis	5	515	2.33	2.95, 0.114	32	0.84 (0.70–0.92)	0.88 (0.80–0.94)	7.2 (3.9–13.2)	0.18 (0.09–0.36)	40 (13–121)	0.93 (0.90–0.95)
Cirrhosis	5	579	2.82	33.65, < 0.001	94	0.86 (0.67–0.95)	0.95 (0.77–0.99)	18.0 (3.5–93.5)	0.15 (0.06–0.37)	123 (25–610)	0.95 (0.93–0.97)

HBV, hepatitis B virus; HCV, hepatitis C virus; NAFLD, nonalcoholic fatty liver disease; NASH, nonalcoholic steatohepatitis; AIH, autoimmune hepatitis; PBC, primary biliary cirrhosis; BA, biliary atresia; COI, cutoff index; CI, confidence interval; PLR, positive likelihood ratio; NLR, negative likelihood ratio; DOR, diagnostic odds ratio; AUSROC, area under the summary receiver operating characteristic curve.

^a^Other etiologies include AIH, BA, PBC and mixed etiologies.

### Predictive accuracy of WFA+-M2BP versus non-invasive indicators for grading liver fibrosis

Due to limited number of studies containing the information of other non-invasive indicators in mild fibrosis, we compared WFA+-M2BP with other non-invasive indicators for predicting significant fibrosis, advanced fibrosis and cirrhosis. As shown in Table 4, for significant fibrosis, the AUSROC of WFA+-M2BP (0.79) was only greater than that of AST/ALT (0.74, P = 0.048). For the detection of advanced fibrosis, WFA+-M2BP yielded AUSROC (0.82) similar to those of APRI (0.78, P = 0.113), FIB-4 (0.79, P = 0.235), HA (0.82, P = 1.0), and FibroScan (0.81, P = 0.831). Greater AUSROC of WFA+-M2BP was only observed when it was compared with AST/ALT (0.67, P < 0.001) and PLT (0.69, P < 0.001). However, when determining cirrhosis, WFA+-M2BP surpassed 4 indicators (WFA+-M2BP = 0.88; APRI = 0.79, P < 0.001; FIB-4 = 0.83, P = 0.034; AST/ALT = 0.79, P < 0.001, PLT = 0.83, P = 0.021) except for HA and FibroScan (HA = 0.88, P = 1.0; FibroScan = 0.87, P = 0.644). Those results indicated that WFA+-M2BP owned the best performance for diagnosing cirrhosis by exceeding most of the widely used indicators.

**Table 4:** AUSROC values of seven non-invasive indicators for predicting significant fibrosis, advanced fibrosis and cirrhosis.

Indicators	Significant fibrosis					Advanced fibrosis					Cirrhosis				
	Number of studies	Number of patients	AUSROC (95% CI)	Z value	P value	Number of studies	Number of patients	AUSROC (95% CI)	Z value	P value	Number of studies	Number of patients	AUSROC (95% CI)	Z value	P value
WFA+-M2BP	20	3,602	0.79 (0.75–0.82)			28	4,427	0.82 (0.78–0.85)			21	3,449	0.88 (0.85–0.91)		
APRI	9	1,563	0.77 (0.74–0.81)	0.79	0.428	18	2,552	0.78 (0.74–0.81)	1.58	0.113	11	1,720	0.79 (0.75–0.82)	3.83	< 0.001
FIB-4	10	1,723	0.76 (0.72–0.79)	1.19	0.235	18	2,512	0.79 (0.75–0.82)	1.19	0.235	11	1,616	0.83 (0.79–0.86)	2.13	0.034
AST/ALT	5	746	0.74 (0.70–0.77)	1.98	0.048	8	960	0.67 (0.63–0.71)	5.53	< 0.001	4	444	0.79 (0.75–0.82)	3.83	< 0.001
HA	6	1,230	0.83 (0.79–0.86)	1.58	0.113	15	2,332	0.82 (0.79–0.85)	0.00	1.000	10	1,608	0.88 (0.85–0.91)	0.00	1.000
PLT	7	1,177	0.73 (0.68–0.76)	0.50	0.619	14	1,971	0.69 (0.65–0.73)	5.53	< 0.001	8	1,221	0.83 (0.80–0.86)	2.31	0.021
FibroScan	1	165	0.83 (0.77–0.89)	1.13	0.259	1	151	0.81 (0.71–0.88)	0.21	0.831	2	235	0.87 (0.84–0.90)	0.46	0.644

WFA+-M2BP, wisteria floribunda agglutinin-positive Mac-2-binding protein; APRI, Aspartate aminotransferase-to-platelet ratio index; FIB-4, Fibrosis-4 index; AST/ALT, AST to ALT ratio; HA, hyaluronic acid; PLT, platelet count; AUSROC, area under the summary receiver operating characteristic curve; CI, confidence interval.

### Diagnostic accuracy of WFA+-M2BP for the prediction of HCC

For the prediction of HCC, a total of 8 studies with 2,240 participants were selected (Table 2). Among them, 4 studies reported the occurrence of HCC after antiviral treatment or HBeAg seroconversion^49,50,55,56^, one study discussed the reoccurrence of HCC after curative resection^54^, and 3 studies focused on the development of HCC^51–53^. The WFA+-M2BP levels here were pretreatment or basal levels. As several studies described the diagnostic information of APRI, FIB-4, and AFP, we compared the diagnostic accuracies of WFA+-M2BP with these three indicators for HCC. There was no significant threshold effect in included studies (r = − 0.7, P = 0.49). However, significant heterogeneity was observed (Table 5). In addition, among all the indicators, WFA+-M2BP yielded the highest pooled sensitivity (0.77, 95% CI 0.60–0.89) which surpassed APRI, FIB-4 and AFP. Although AFP had the highest pooled specificity (0.94, 95% CI 0.82–0.98), the AUSROCs of WFA+-M2BP and AFP were very similar (P = 0.671).

**Table 5:** Meta-analyses results of four non-invasive markers for diagnosing HCC.

Indicators	Number of studies	Number of patients	Heterogeneity		Sensitivity (95% CI)	Specificity (95% CI)	Weighted Mean cutoff	AUSROC (95% CI)	Z value	P value
			Q value, P value	I^2^ (%)						
WFA+-M2BP	8	2,240	95.18, < 0.001	98	0.77 (0.60–0.89)	0.80 (0.71–0.86)	1.55 (COI)	0.85 (0.82–0.88)		
APRI	1	119	NA	NA	0.67	0.81	0.55	0.74	6.99	< 0.001
FIB-4	1	119	NA	NA	0.67	0.81	2.95	0.78	4.77	< 0.001
AFP	3	1,340	63.00, < 0.001	97	0.61 (0.42–0.77)	0.94 (0.82–0.98)	18.54 (ng/mL)	0.84 (0.80–0.87)	0.43	0.671

HCC, hepatocellular carcinoma; WFA+-M2BP, wisteria floribunda agglutinin-positive Mac-2-binding protein; APRI, aspartate aminotransferase-to-platelet ratio index; FIB-4, fibrosis-4 index; AFP, α-fetoprotein; AUSROC, area under the summary receiver operating characteristic curve; CI, confidence interval; NA, not available.

### Publication bias and sensitivity analysis

As displayed in Supplementary Fig. 3, Deek’s funnel plots were almost symmetric for studies that reported mild liver fibrosis, significant fibrosis, and advanced fibrosis (P values = 0.1, 0.33, and 0.09, respectively), suggesting no evidence of publication bias. However, a significant publication bias was observed in studies on cirrhosis (P = 0.03). For studies on the prediction of HCC, there was no publication bias (P = 0.83) (Supplementary Fig. 4).

Through sensitivity analysis, we observed outlier studies existed in each stage of liver fibrosis (Supplementary Fig. 5). Surprisingly, after the removal of outlier studies, the heterogeneity in mild fibrosis disappeared (Supplementary Table 1) and the publication bias in studies on cirrhosis was diminished (Supplementary Fig. 6). However, Supplementary Table 1 indicated that the summary results were not significantly affected by individual studies. Also, as displayed in Supplementary Fig. 7, no outlier study was found in HCC.

## Discussion

WFA+-M2BP is a serum glycobiomarker that is receiving growing attentions. It had been reported that the elevated WFA+-M2BP level was associated with the risk of HCC^57–59^, the loss of HBeAg in chronic hepatitis B patients^60^, and the severity of liver fibrosis^61,62^. In our meta-analysis, we evaluated 36 articles in total, and explored the predictive accuracy of WFA+-M2BP for distinguishing liver fibrosis stages and HCC by comparing with diverse non-invasive indicators. Our results suggested WFA+-M2BP possessed satisfactory diagnostic accuracy for predicting cirrhosis and moderate diagnostic performance for detecting mild fibrosis, significant fibrosis, advanced fibrosis and HCC. The AUSROC of WFA+-M2BP was equivalent to HA and FibroScan for assessing cirrhosis, and similar to AFP for diagnosing HCC.

Previously, literatures on the diagnostic performance of WFA+-M2BP in different stages of liver fibrosis were controversial. Zou et al.^26^ reported WFA+-M2BP was useful to assess early stages of liver fibrosis, and Ura et al.^36^ showed WFA+-M2BP was more accurate in diagnosing significant fibrosis than advanced fibrosis. In contrast, several other studies indicated that WFA+-M2BP had the strongest ability to predict cirrhosis^37,62^. In our meta-analysis, we found that the overall AUSROCs of WFA+-M2BP for identifying mild fibrosis, significant fibrosis, advanced fibrosis and cirrhosis were 0.75, 0.79, 0.82 and 0.88, respectively. For cirrhosis, WFA+-M2BP reached the highest pooled sensitivity and specificity at 0.77 and 0.86. Since it is widely accepted that AUC between 0.85 and 0.90 is as good as liver biopsy for staging fibrosis^63^, our study underscores the notion that WFA+-M2BP could serve as a surrogate biomarker for biopsy when diagnosing cirrhosis.

Interestingly, WFA+-M2BP exhibited different predictive accuracies for staging liver fibrosis caused by different etiologies in CLDs. In our study, for patients with AIH, BA, PBC or mixed etiologies, WFA+-M2BP exhibited excellent performance for distinguishing significant fibrosis, advanced fibrosis and cirrhosis. In general, WFA+-M2BP had lower accuracy in HBV-infected patients than in patients with HCV infection, NAFLD or NASH. Our conclusion was consistent with a previous meta-analysis^23^. However, our study was more extensive, as we included more publications, had different subgroup setups, and used different models for the calculation of pooled results. WFA+-M2BP has the potential to reflect hepatic fibrosis as hepatic stellate cells (HSCs) are the source of WFA+-M2BP and its level is closely associated with α-smooth-muscle actin (αSMA) expression^19,21^. However, HBV‐positive patients are more likely to experience quiescent hepatic inflammation, and HBV-related cirrhosis had large regenerative nodules and thin fibrous septa^23,27^. As a result, as shown in Table 3, minor changes of WFA+-M2BP optimal cutoffs in each liver fibrosis stage of HBV-infected patients may lead to low diagnostic accuracy. Whether better predictive performance of WFA+-M2BP is related to more severe liver damage or inflammation response, or more activation of HSCs, will need to be investigated by further studies. Here, we suggest that individual cutoff value of WFA+-M2BP should be used to stage liver fibrosis of different etiology.

Overall, WFA+-M2BP was not better than other non-invasive indicators for predicting significant fibrosis and advanced fibrosis. However, for assessing cirrhosis, the diagnostic accuracy of WFA+-M2BP was equivalent to HA and FibroScan and superior to four markers (i.e., APRI, FIB-4, AST/ALT, and PLT). First, our meta-analysis study revealed similar AUSROC values of APRI and FIB-4 in each fibrosis stage (significant fibrosis: 0.7407 and 0.7844, advanced fibrosis: 0.7347 and 0.8165, and cirrhosis: 0.7268 and 0.8448, respectively)^64^, indicating the reliability of our analysis. Second, we observed the similar result as a previous study, which reported that HA was more efficient than APRI and FIB-4 for fibrosis staging^63,65^. Although studies claimed that FibroScan could offer more promising results than HA for staging both early hepatic fibrosis and cirrhosis^66,67^, we were not able to tell the difference between FibroScan and HA in this study due to limited sample size. Since the accuracy of FibroScan is strongly influenced by disease etiology^68,69^, additional studies stratified by etiology will be necessary to draw the conclusion.

As M2BP has been shown to promote cancer progression, WFA+-M2BP may potentially be used to predict HCC development^18^. In our current study, WFA+-M2BP was superior to APRI and FIB-4 and equivalent to AFP for diagnosing HCC. Currently, whether AFP should be used for routine surveillance of HCC is controversial due to its limited sensitivity in early detection. In our study, WFA+-M2BP had a higher sensitivity (0.77) and a lower specificity (0.80) when it was compared with AFP (sensitivity = 0.61 and specificity = 0.94). This finding indicated the possibility of improving the diagnosis of HCC if WFA+-M2BP and AFP are used together. Besides, it had been reported the posttreatment level of WFA+-M2BP could nicely reflect HCC development for patients underwent anti-viral therapy by reaching a high sensitivity, specificity and AUROC (0.875, 0.939 and 0.973)^49^. As a result, combined diagnostic model or posttreatment detection of WFA+-M2BP might be helpful for the prediction of HCC.

It should be noted that there are limitations in our study: (1) Due to the limited study number, we could not evaluate the predictive accuracy of WFA+-M2BP in mild fibrosis stratified by etiology; (2) This meta-analysis is a pilot study which compared the performance of multiple indicators by analyzing results from literatures on WFA+-M2BP. We suggested future study could be performed to investigate the comparison between WFA+-M2BP and a certain non-invasive indicator for staging liver fibrosis or predicting HCC in a certain disease by analyzing more literatures from multiple databases; (3) More high-quality studies are needed for analysis. Our quality assessment demonstrated that the existence of moderate risk of bias was mainly due to the awareness of reference standard result before conducting the index test. Also, significant heterogeneities existed in studies regarding each stage of liver fibrosis and HCC. And we found that the age of the participants, male proportion, etiology, and certain outlier studies might be the source of these heterogeneities. Besides, we noticed obvious publication bias existed in studies on cirrhosis, although this bias could be diminished after the removal of three outlier studies. (4) We conducted this diagnostic meta-analysis and emphasized the diagnostic performance of WFA+-M2BP in liver fibrosis and HCC, without elaborating on the outcomes of patients with different basal levels of serum WFA+-M2BP.

In conclusion, our meta-analysis demonstrated that WFA+-M2BP could be used as a surrogate biomarker for liver biopsy to diagnose cirrhosis in chronic liver diseases. It showed modest accuracy for identifying early stage of liver fibrosis and HCC. Compared with other non-invasive indicators, the predictive performance of WFA+-M2BP was similar to HA and FibroScan in assessing cirrhosis, but was equivalent to AFP in HCC. As the accuracy of WFA+-M2BP was strongly influenced by the etiology of disease, individual cutoff value was suggested to be applied in certain etiology.

## Methods

### Literature search strategy

We performed this meta-analysis according to the Cochrane Handbook for Systematic Reviews of Diagnostic Test Accuracy^70^. Literatures published before September 22, 2019 in Pubmed, Web of Science, EMBASE, the Cochrane Library, and grey literature database including OpenGrey (https://www.opengrey.eu) were searched. The search terms included “Wisteria floribunda agglutinin-positive Mac-2-binding protein or WFA+-M2BP or M2BPGi or Mac-2-binding protein glycosylation isomer” and “fibrosis or cirrhosis” or “hepatocellular carcinoma or liver cancer”.

### Inclusion and exclusion criteria

Inclusion criteria were: (1) study objects contained patients with liver fibrosis or hepatocellular carcinoma caused by various etiologies; (2) studies used liver biopsy as gold standard to measure the severity of liver fibrosis; (3) studies used typical imaging techniques and/or histopathology to diagnose HCC; (4) studies employed WFA+-M2BP and may also include APRI, FIB-4, aspartate aminotransferase to alanine aminotransferase ratio (AST/ALT), HA, PLT, FibroScan stiffness value or AFP to predict liver fibrosis or HCC; and (5) data on true-positive (TP), true-negative (TN), false-positive (FP), false-negative (FN) results were reported separately or could be calculated from the article.

Studies as follows were excluded: (1) duplicate studies; (2) non-experimental studies such as reviews, letters, clinical trials, correspondences, comments, case reports, and patents; (3) studies published in a language other than English; (4) non-human subjects; or (5) the relevant data were inaccessible or unclear.

### Data extraction and quality assessment

Firstly, we employed the Quality Assessment of Diagnostic Accuracy Studies-2 (QUADAS-2)^71^ to assess the quality of the selected studies independently. Next, the following information were extracted: first author’s name, year of publication, region, number of patients, type of biomarkers, age, proportion of male, etiology, histological system, blind method, liver biopsy length, interval between biopsy and blood test, patient number in different fibrosis stages, WFA+-M2BP cutoff values, and TP, TN, FP, and FN results. Besides, Metavir, Brunt, Batts and Ludwig or Revised Inuyama stages ≥ F1, ≥ F2, ≥ F3, and F4 were defined as mild fibrosis, significant fibrosis, advanced fibrosis, and cirrhosis, respectively.

### Statistical analyses

We calculated 2 × 2 tables and performed QUADAS-2 quality assessment by using Review Manager 5.2 (The Nordic Cochrane Centre, Copenhagen, Denmark). Besides, we employed bivariate random effects model to conduct statistical analysis. Thus, “Midas” module in Stata version 14.2 (StataCorp, College Station, TX) was used to summarize test accuracy: pooled sensitivity, specificity, positive likelihood ratio (PLR), negative likelihood ratio (NLR), diagnostic odds ratio (DOR), the area under the summary receiver operating characteristic curve (AUSROC). Z test was adopted to compare AUSROCs of WFA+-M2BP and other indicators. Diagnostic threshold effect would exist if the Spearman correlation coefficient > 0 and P < 0.05^72^. If inconsistency index (I^2^) ≥ 50% or P < 0.05 was observed, it suggested significant heterogeneity^73^. In that case, we would conduct meta-regression analysis, which evaluated potential factors to determine covariates. In joint model, factors with P < 0.1 was considered the potential source of heterogeneity^74,75^. Furthermore, publication bias was determined by using Deeks’s funnel plot, and P < 0.05 indicated possible bias^76^. Finally, we carried out sensitivity analysis to measure the robustness of the summary results.

## Supplementary information

Supplementary file
