# Supplementary material for: Wisteria floribunda agglutinin-positive Mac-2-binding protein as a diagnostic biomarker in liver cirrhosis: an updated meta-analysis
Source: Sci Rep. 2020 Jun 29;10:10582. doi: 10.1038/s41598-020-67471-y (PMC7324360; doi:10.1038/s41598-020-67471-y)
Supplement: Supplementary file 1 — Supplementary file [file 41598_2020_67471_MOESM1_ESM.pdf]

Wisteria floribunda agglutinin-positive Mac-2-binding protein as a diagnostic biomarker in liver cirrhosis: An updated meta-analysis

Shu Feng[1], Zhonghao Wang[1], Yanhua Zhao[1], Chuanmin Tao[1]*

[1]Department of Laboratory Medicine, West China Hospital, Sichuan University, Chengdu, Sichuan 610041, China

* Corresponding author: Chuanmin Tao

Tel: +86-28-85422612

Fax: +86-28-85582944

Email address: taocm@scu.edu.cn

Supplementary materials

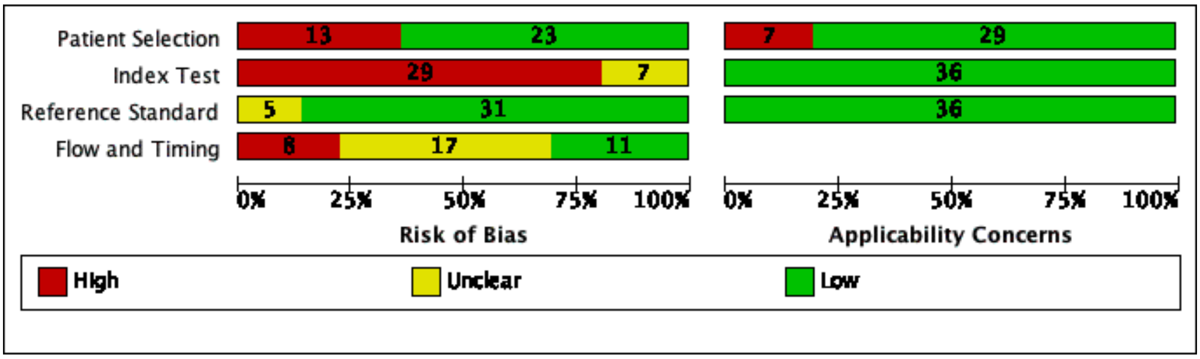

Supplementary Figure 1. Graph of risk of bias and applicability concerns of overall quality assessment for all included studies by QUADAS-2 tool.

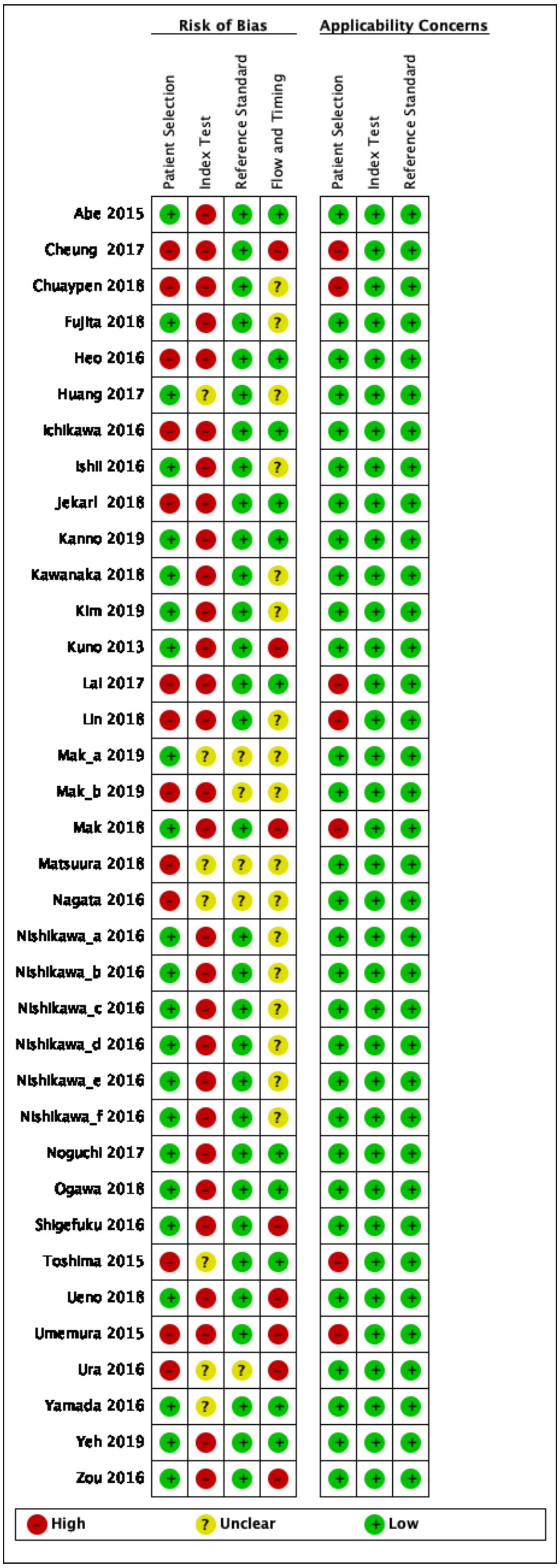

Supplementary Figure 2. Summary of risk of bias and applicability concerns of overall quality assessment for all included studies by QUADAS-2 tool.

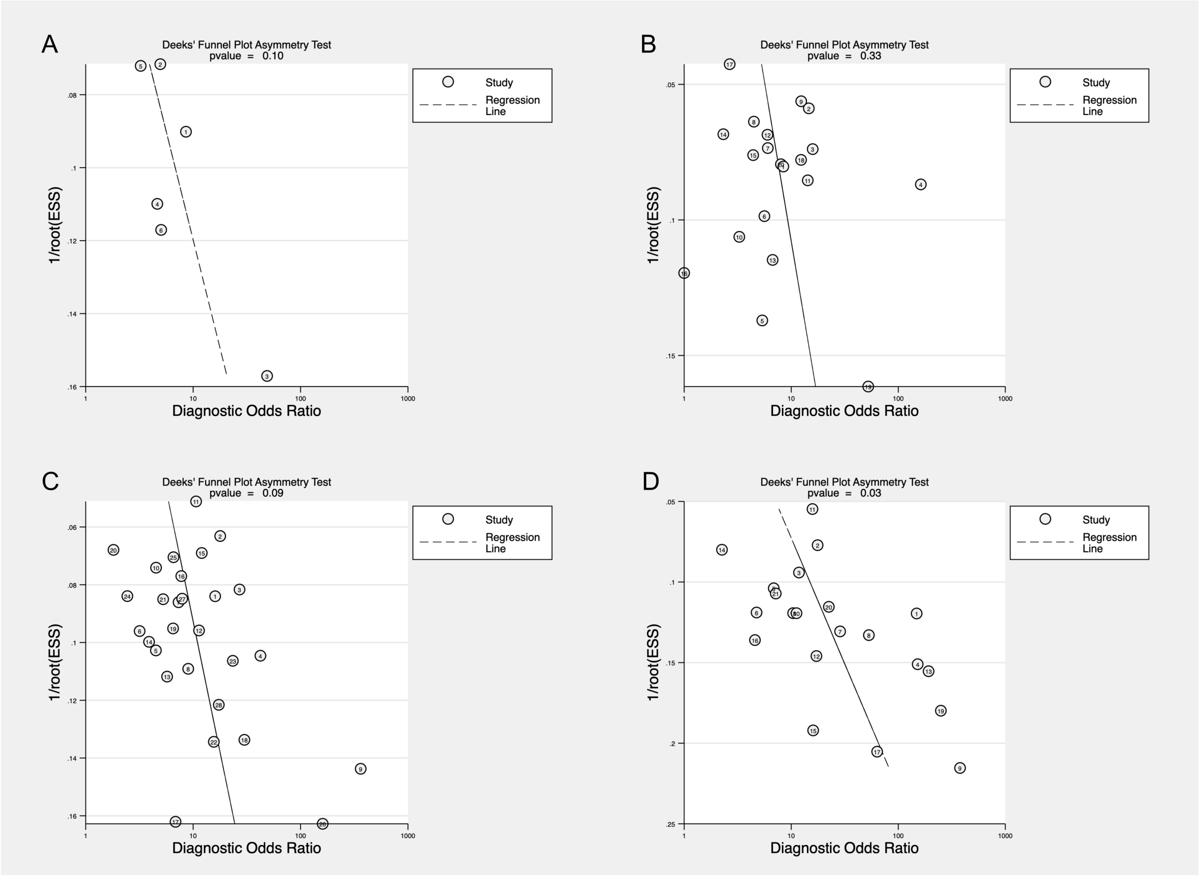

Supplementary Figure 3. Publication bias analysis of WFA+ -M2BP for the prediction of liver fibrosis. A: Mild fibrosis. B: Significant fibrosis. C: Advanced fibrosis. D: Cirrhosis.

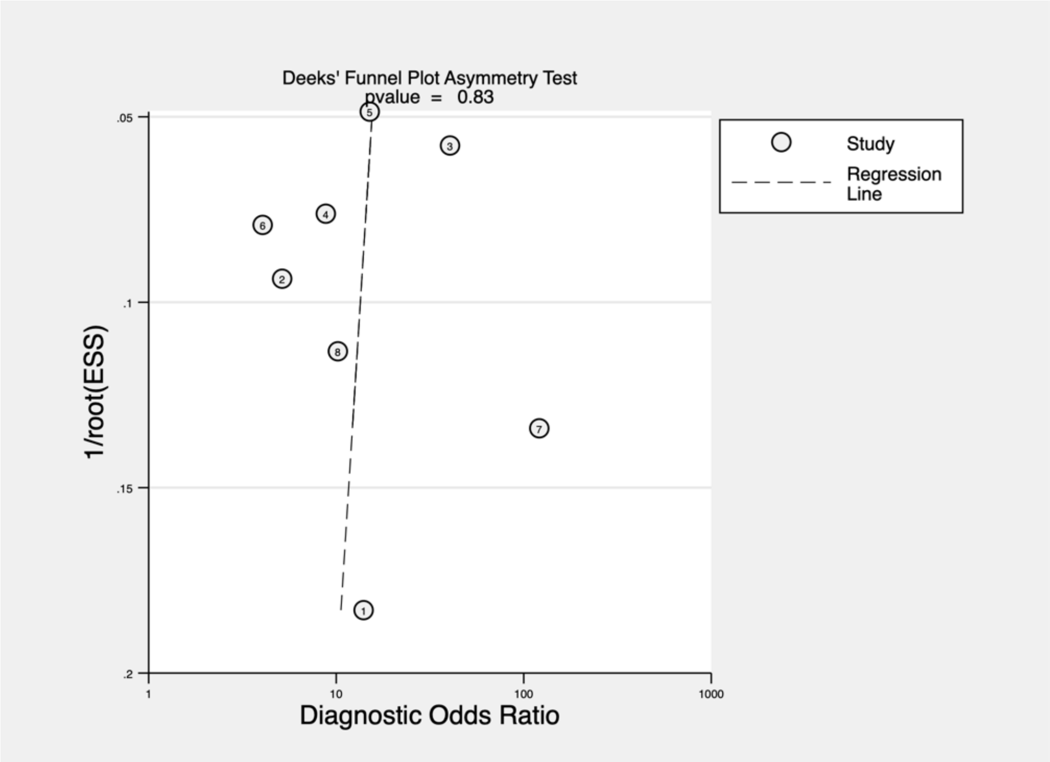

Supplementary Figure 4. Publication bias analysis of WFA+ -M2BP for the identification of HCC.

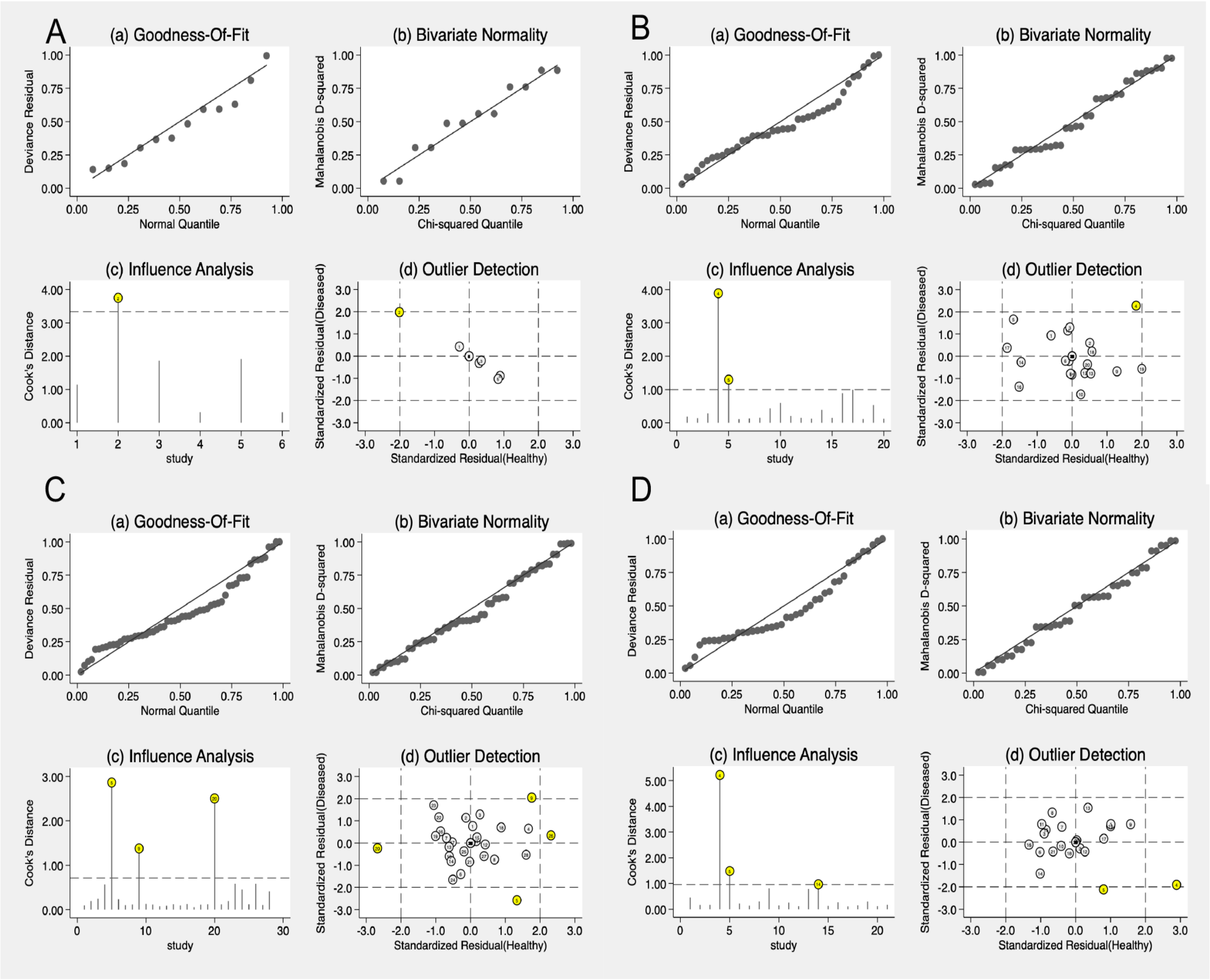

Supplementary Figure 5. Sensitivity analysis of WFA+ -M2BP for the prediction of liver fibrosis. A: Mild fibrosis. B: Significant fibrosis. C: Advanced fibrosis. D: Cirrhosis.

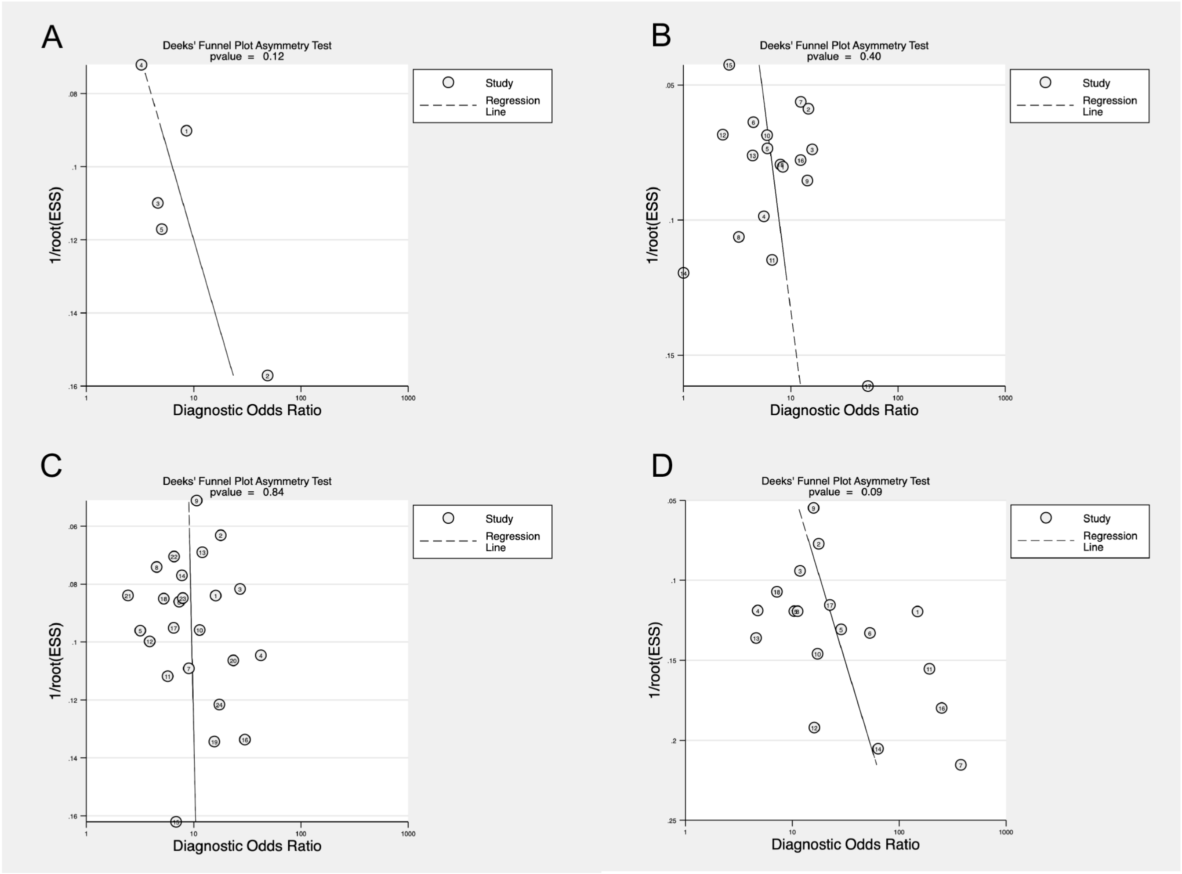

Supplementary Figure 6. Publication bias analysis of WFA+ -M2BP for the prediction of liver fibrosis after removing outlier studies. A: Mild fibrosis. B: Significant fibrosis. C: Advanced fibrosis. D: Cirrhosis.

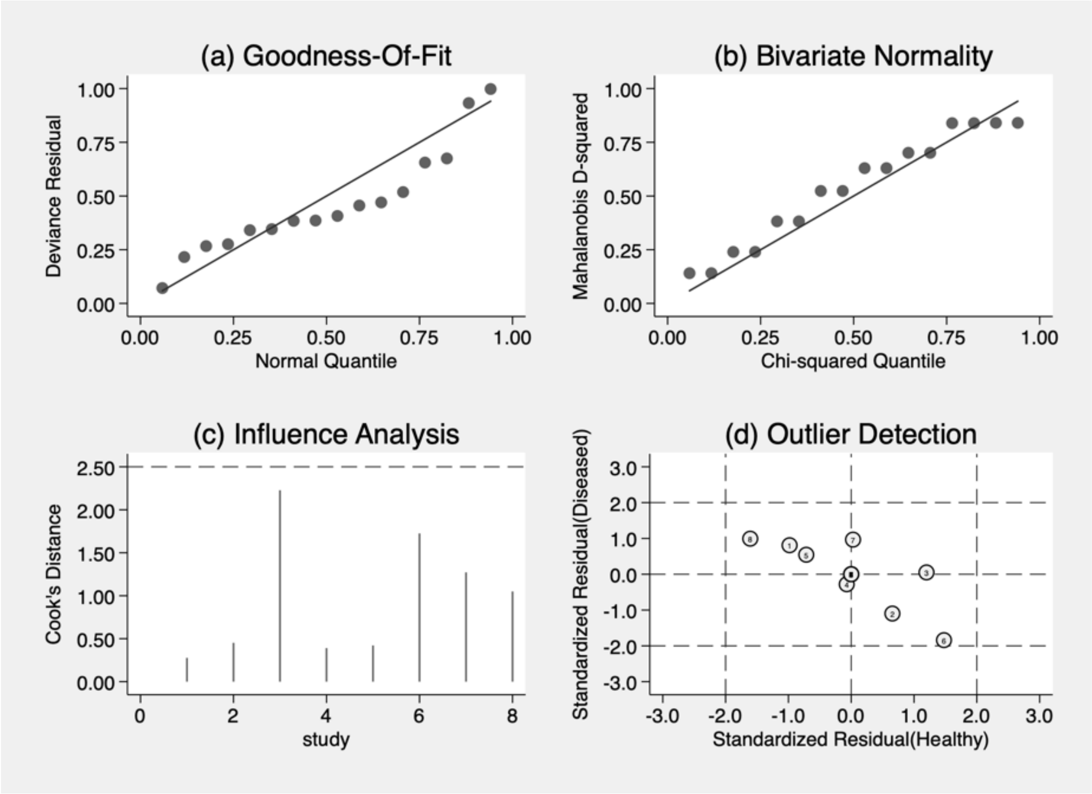

Supplementary Figure 7. Sensitivity analysis of WFA+ -M2BP for the diagnosis of HCC.

Fibrosis stages	Outlier studies	Overall Heterogeneity		Pooled sensitivity (95% CI)	Pooled specificity (95% CI)	Pooled AUSROC (95% CI)
		Q value, P value	I^2 (%)			
Mild fibrosis	NA	23.11, <0.001	91	0.70 (0.62-0.77)	0.68 (0.57-0.78)	0.75 (0.71-0.78)
Significant fibrosis	NA	94.75, <0.001	98	0.71 (0.65-0.76)	0.75 (0.69-0.81)	0.79 (0.75-0.82)
Advanced fibrosis	NA	50.32, <0.001	96	0.75 (0.69-0.79)	0.76 (0.72 - 0.80)	0.82 (0.78 - 0.85)
Cirrhosis	NA	64.79, <0.001	97	0.77 (0.69-0.84)	0.86 (0.79 - 0.90)	0.88 (0.85 - 0.91)
**After removal of outlier studies**						
Mild fibrosis	Toshima 2015[1]	0.418, 0.406	0	0.67 (0.61-0.72)	0.74(0.66-0.81)	0.77 (0.73-0.81)
Significant fibrosis	Umemura 2015[2], Heo 2016[3]	87.82, <0.001	98	0.68 (0.63-0.72)	0.75 (0.60-0.80)	0.77 (0.73-0.80)
Advanced fibrosis	Heo 2016[3], Nishikawa_b 2016[4], Huang 2017[5], Ueno 2018[6]	14.25, <0.001	86	0.75 (0.70-0.79)	0.75 (0.72-0.78)	0.81 (0.78-0.84)
Cirrhosis	Umemura 2015[2], Heo 2016[3], Huang 2017[5]	13.26, 0.001	85	0.82 (0.77-0.87)	0.84 (0.78-0.88)	0.89 (0.86-0.92)

Supplementary Table 1. Overview of meta-analyses results before and after removal of outlier studies in liver fibrosis. Abbreviations: CI, confidence interval; AUSROC, area under the summary receiver operating characteristic curve; NA, not available.

References:

1 Toshima, T. *et al.* A novel serum marker, glycosylated Wisteria floribunda agglutinin-positive Mac-2 binding protein (WFA(+)-M2BP), for assessing liver fibrosis. *J Gastroenterol* **50**, 76-84, doi:10.1007/s00535-014-0946-y (2015).

2 Umemura, T. *et al.* Serum Wisteria floribunda agglutinin-positive Mac-2-binding protein level predicts liver fibrosis and prognosis in primary biliary cirrhosis. *Am J Gastroenterol* **110**, 857-864 (2015).

3 Heo, J. Y. *et al.* Use of Wisteria Floribunda Agglutinin-Positive Human Mac-2 Binding Protein in Assessing Risk of Hepatocellular Carcinoma Due to Hepatitis B Virus. *Medicine* **95**, doi:10.1097/md.0000000000003328 (2016).

4 Nishikawa, H. *et al.* Impact of serum Wisteria floribunda agglutinin positive Mac-2-binding protein and serum interferon-gamma-inducible protein-10 in primary biliary cirrhosis. *Hepatol Res* **46**, 575-583 (2016).

5 Huang, C. I. *et al.* Serum Wisteria floribunda agglutinin-positive Mac-2-binding protein expression predicts disease severity in chronic hepatitis C patients. *Kaohsiung J Med Sci* **33**, 394-399, doi:10.1016/j.kjms.2017.05.017 (2017).

6 Ueno, T. *et al.* Clinical implications of serum Mac-2-binding protein (M2BPGi) during regular follow-up of patients with biliary atresia. *Pediatr Surg Int* **34**, 1065-1071, doi:10.1007/s00383-018-4317-2 (2018).
